# The using of a piglets as a model for evaluating the dipyrone hematological effects

**DOI:** 10.1186/s12917-016-0891-5

**Published:** 2016-11-25

**Authors:** Artur Burmańczuk, Andrzej Milczak, Tomasz Grabowski, Monika Osypiuk, Cezary Kowalski

**Affiliations:** 1 Department of Pharmacology, Faculty of Veterinary Medicine, University of Life Sciences, Akademicka 12, 20-033 Lublin, Poland; 2 Department and Clinic of Animal Internal Diseases, Sub-Department of Companion Animal Internal Medicine, Faculty of Veterinary Medicine, University of Life Sciences, Głęboka 30, 20- 612 Lublin, Poland; 3 Polpharma Biologics, Trzy lipy 3, 80-172 Gdańsk, Poland

**Keywords:** Metamizole, Dipyrone, Hematology, Piglet

## Abstract

**Background:**

Dipyrone (MET, metamizole) is a non-steroidal anti-inflammatory drug commonly used both in human and in veterinary medicine. After oral administration, is broken down rapidly to metabolites which largely retain the activity of the parent drug. Its metabolites have analgesic, antipyretic and anti-inflammatory effects.

**Results:**

The subjects were eight healthy male Large White post-suckling piglets, weighing between 5.0 to 7.4 kg, of ages 35 ± 10 days. The animals were administered MET (100 mg/kg) by an intramuscular (I.M.) injection. The study calculated the value of several hemorheological parameters. Significant impact of MET treatment (*p* < 0.05) was proven in case: activated partial thromboplastin time; ratio of activated partial thromboplastin time; hemoglobin; hematocrit; mean corpuscular hemoglobin; mean corpuscular volume; red blood cells volume; white blood cells volume; prothrombin time index.

**Conclusions:**

In summation, our observations suggest that a piglet model is useful for studying the impact of MET on hemorheological parameters.

**Electronic supplementary material:**

The online version of this article (doi:10.1186/s12917-016-0891-5) contains supplementary material, which is available to authorized users.

## Background

Dipyrone (MET, metamizole) is a non-steroidal anti-inflammatory drug commonly used both in human and in veterinary medicine. In some countries dipyrone is banned because of serious adverse effect [1]. But still the drug is available in many countries depending on registration agency [2]. Like most anti-inflammatory drugs, MET is not without adverse effects. MET, after oral administration, is broken down rapidly to metabolites which largely retain the activity of the parent drug. These, 4 aminoantipirin (4AA) and 4 methylaminoantipirin (4MAA), the pharmacokinetics of which were described previously by Burmańczuk [3], have analgesic, antipyretic and anti-inflammatory effects [4]. Still, the sum of the side effects related to MET administration includes activities whose core has an effect on textured blood components (neutrophil activation), and MET also has impact on the functioning of the immune system reactions (IgE mediated and specific T lymphocytes, CD8^+^) [5, 6]. What is more, the use of the basophil-activation test has revealed that the MET metabolites activate basophils in a specific way in patients who are hypersensitive to MET [7]. Still, agranulocytosis following administration of MET in humans is a very rare phenomenon, however, it has been confirmed that a significant fall in the number of leukocytes after administration of MET comes about in individual cases [8]. Such agranulocytosis in humans is at a level of 2.4–14.5 ppm [9]. MET, at higher doses, also affects catalase levels in human red blood cells (RBC) [10]. Furthermore, dipyrone inhibits H_2_O_2_ forced erythrocytic membrane lipid peroxidation [10], this means that it has a significant impact on the physiology of the oxidation processes in human RBC. Yet, as up to 30% of all patients are hypersensitive to pyrazolones after the administration of MET (reacting through anaphylactic shock), the impact of MET on hemorheological parameters has not been fully explained [5–11].

For many years, the pig model has been one of the more interesting ways of testing drugs destined for human administration. Indeed, such a model is widely used in preclinical studies, particularly with respect to the employment of miniature pigs [12]. It is important to note that the pig and piglets is also considered as being one of the best non-rodent test-subjects in preclinical therapeutic human monoclonal antibody studies [13]. It is thought that large animals such as the pig, rather than small animals, may be significantly better models in the study of medicines destined for humans. It can be said, then, that the pig model better reflects human drug metabolism characteristics [14]. Hence, current study verified the use of piglets as test system which may reflect changes in hematological parameters after a single I.M. injection of MET.

## Methods

For presented study dipyrone was selected as an agent that inhibits the platelet aggregation [15]. Moreover single-dose dipyrone have similar efficacy to other analgesics used in postoperative pain [16]. Single dose study was proposed due to the fact that single dose analysis could indicate the choice of dosing interval and dose [17]. Consequently the studies with multiple dose should be preceded by single dose study to avoid mistakes in optimization of multiple dose studies.

### Animals

The subjects were eight healthy male Large White post-suckling piglets, weighing between 5.0 to 7.4 kg, of ages 35 ± 10 days. The piglets were deprived of food for 8 h prior to the commencement of the experiment, while water was available *ad libitum*. The animals were administered MET (100 mg/kg; Biovetalgin, 500 mg/mL, Biowet, Drwalew, Poland) by an I.M. injection.

In humans, the standard dipyrone dosage is 0.5 to 1.0 g, up to four times a day [4]. Our study determined that the typical dose which is given to piglets weighing about 5 kg was similar to that which is commonly administered to humans weighing about 70 kg (0.5 g per animal). After I.M. drug administration, blood was sampled from the jugular vein (2 mL) at intervals of 0 before dosing (control) 0.25, 0.50, 0.75, 1, 2, 4, 6, 8, 10, 12, 24, 48, 72 h into heparinized tubes, by vacutainer (BD Vacutainer® Safety- Lok). The blood plasma 4MAA and 4AA concentrations were subsequently analyzed by high pressure liquid chromatography [18]. The study protocol was approved by the ethics committee of the University of Life Sciences, Lublin (84/2015).

### Hemorheological parameters

The taken blood samples were analyzed for basic indicators of blood counts (white blood cells – WBC). Moreover, the following parameters were determined using an automated hematology analyzer – Abacus Junior Vet (Diatron Group, Hungary): APTT – activated partial thromboplastin time; APTTratio – ratio of activated partial thromboplastin time; Hb – hemoglobin; Hct – hematocrit; MCH – mean corpuscular hemoglobin; MCV – mean corpuscular volume; RBC – red blood cells volume; WBC – white blood cells volume; PT – prothrombin time index; MCHC – mean corpuscular hemoglobin concentration; Plt – platelets.

Platelet function testing was performed by way of employing an automated analyzer - PFA-200 INNOVANCE® Analyzer (Healthineers Siemens, Germany), to assess rates of platelet activation: ADP-dependent and COX-1 dependent pathway. The study used citrated whole blood analysis via two types of cartridges dubbed with the corresponding agonist of platelet activation: a Collagen/ADP Test Cartridge (collagen type I with adenosine diphosphate) and Collagen/EPI Test Cartridge (type I collagen and epinephrine bitartrate) (Dade Behring, Germany). The study was performed within 15 min of the collection of blood and platelet activation was measured by quantity of time needed to complete closure of the micro-holes of the cartridge membrane and the cessation of blood flow – the so-called ‘occlusion time’ (CT – closure time). Herein, a shorter CT means a higher degree of platelet activity, whereas an elongated CT indicates their lower activity [19–23].

The condition (status) of plasma coagulation was assessed by way of the maintenance of the hemostasis tests: the prothrombin time and APTT. Fibrinogen concentration was also determined by prothrombin time dependent method using thromboplastin [24, 25]. The study employed an automatic optical coagulometer Bioksel 6000 (Bio-Ksel, Poland). The reagents used were supplied by the same company: for the determination of prothrombin time and fibrinogen concentration - Bio-Ksel PT plus, and for APTT measurement - Bio-Ksel System APTTs. All assays were performed in fresh platelet poor citrated plasma, separated from the citrated blood no more than 1 h from (after) its collection. Blood spinning (centrifugation) was carried out at 21 °C at 1500 × g. For calibration the coagulometer used a freeze-dried human standard plasma (Bio-Ksel, Poland). The results for PT and APTT are given in seconds, and the PT and international normalized ratio for prothrombin time and ratio for APTT were calculated [19, 20, 24, 25].

### Pharmacodynamic, pharmacokinetics and statistical analysis

The pharmacodynamics calculations were performed using Phoenix® WinNonlin® 6.4 software (Certara L.P., US), while statistical analyses were carried out by way of GraphPad Prism® 6.01 software (GraphPad Software Inc., US). The aforementioned utilized pharmacokinetic parameters were those described earlier by Burmańczuk et al [3]. The pharmacodynamic calculations were based on the area under the effect curve (AUEC) analysis. This is calculated via the linear trapezoidal approach, and it is ascertained firstly for its baseline (B) in a range beginning 36 h prior to administration and extending to a time equal to zero just prior to MET administration, and then for a curve relating the effect after drug administration (A) in the range of 0–72 h after MET administration. Herein, the time (36 h↔0 h↔72 h) ratio of AUEC effect/baseline (A/B) was produced using the formula. Differences between AUEC baseline (B/36) and the curve relating the effect (A/72) were considered statistically significant at *p* < 0.05. The level of significance was determined by way of applying the paired Student’s *t* test.

The study also sought to bring about an understanding of the comprehensive relationship between hemorheological parameters and the concentration of metabolites. In our work, to validate the relationship between the concentrations of metabolites and the hemorheological parameters, the live-one-out (LOO) procedure was utilized [26–30]. Moreover, the squared cross-validated correlation coefficient (*Q* ^2^) parameter and difference between *Q* ^2^ and squared Pearson correlation coefficient (R^2^) was calculated so as to assess the the measure of internal performance and model predictive-ability. In addition, the Total sum of squares was ascertained (SS). Here, a value closer to 0 indicates that the model has a smaller random error, and the fit will be more optimal for prediction. Beyond the aforementioned, we determined the predicted residual sums of squares (PRESS). A lowest value of PRESS indicates a not-over-fitted model. Finally, the difference between the fitting and the predictive ability of the models was analyzed by calculating the difference between the asymptotic squared cross-validated correlation coefficient (*Q* ^2^_asym_) and *Q* ^2^. Herein, a minimal validation criteria is established so to assess whether the adopted model is in line with the requirements of the OECD. In this: *Q* ^2^_asym_ - *Q* ^2^ > 0 < 1 and *Q* ^2^ ≥ 0.65, while *R* ^2^ ≥ 0.85, and *Q* ^2^ - *R* ^2^ < 0 [27, 28]. The only model that meets all the criteria is, at the same time, qualified as being verified positively, as well as being fully predictive.

## Results

**Fig. 1:**
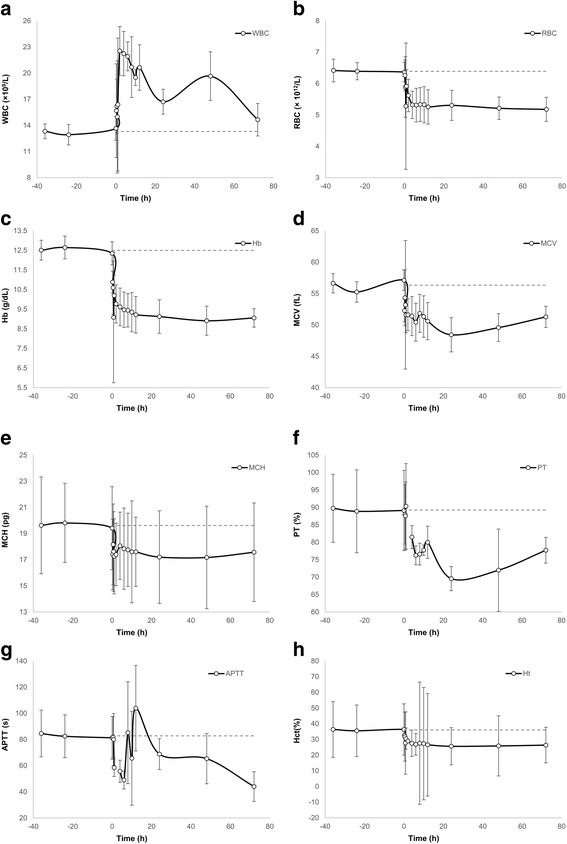
Dynamics of changes in blood parameters after a single I.M. administration of dipyrone in piglets (*n* = 8) at a dose of 100 mg/kg. Changes in: *white* blood cells volume (**a**), *red* blood cells volume (**b**), hemoglobin (**c**), mean corpuscular volume (**d**), mean corpuscular hemoglobin (**e**), prothrombin time index (**f**), activated partial thromboplastin time (**g**), hematocrit (**h**). The period between -36 h and the value 0 represents the baseline - the value before drug administration. APTT – activated partial thromboplastin time (s); APTT_ratio_- ratio of activated partial thromboplastin time; Hb – hemoglobin (g/dL); Hct – hematocrit (%); MCH – mean corpuscular hemoglobin (pg); MCV – mean corpuscular volume (fL); RBC – *red* blood cells volume (× 10^12^ L); WBC – *white* blood cells volume (× 10^9^ L); PT – prothrombin time index (%); dashed line – arithmetic mean of baseline value

The study calculated the value of several hemorheological parameters (Fig. 1; Additional file 1). Table 1 reveals those for which a significant difference was found between AUEC as determined for baseline, and the curve illustrating the effect obtained after MET injection. Herein, Plt after administration showed a marked increase and then a decrease, with Plt peaking at 10 h after MET administration (*p* < 0.05), and the value of Plt 24 h after administration returning to the baseline level. With regard to fibrinogen, after the administration of the drug, our results showed a significant decline, which peaked at 6 h after administration of MET (*p* < 0.05). Fibrinogen levels, however, returned to baseline levels 24 h after administration of MET. It was also confirmed that a significant correlation exists between the level of fibrinogen and WBC (*R* ^2^ = 8.355, *p* < 0.05). The other parameters obtained after administration of MET showed no statistically significant change (baseline: effect).

**Table 1:** Dynamics of changes in blood parameters expressed as minimum, maximum, and area under the curve effect (AUEC) in piglets, after a single I.M. administration of dipyrone at a dose of 100 mg/kg

Blood analysis	Dynamic range of effect minimal to maximal (M; SD)		AUEC (M; SD)		A/B ratio	A/B *p*-value
			A	B		
APTT	113.59; 25.95	45.14; 11.04	4766.87; 542.43	2968.43; 588.85	0.197	0.0207
APTT_ratio_	3.36; 0.77	1.33; 0.33	140.69; 16.01	86.38; 17.14	0.186	0.0259
Hb	12.35; 0.58	9.14; 0.49	628.56; 74.74	450.96; 20.44	0.303	0.0001
Hct	36.32; 1.63	26.64; 1.70	1808.43; 213.89	2586.93; 58.18	0.650	0.0001
MCH	19.65; 0.88	17.58; 0.91	1198.07; 139.58	707.33; 33.62	0.153	0.0046
MCV	58.00; 2.60	51.25; 3.53	3440.34; 406.96	2019.75; 113.64	0.148	0.0052
RBC	6.56; 0.33	5.22; 0.37	362.90; 42.88	229.96; 10.74	0.211	0.0001
WBC	24.67; 2.14	14.71; 1.76	1267.83; 179.64	476.63; 41.72	−0.330	0.0006
PT	95.00; 6.08	77.25; 3.67	5164.33; 699.57	3207.75; 406.96	0.195	0.0076

*M* arithmetic mean, *SD* standard deviation, *A* area under effect curve at time 0 to 72 h (after treatment), *B* area under effect curve at baseline (time between 36 h before treatment and time zero), *A/B* ratio calculated from A and B value, *APTT* activated partial thromboplastin time, *APTT*_*ratio*_ ratio of activated partial thromboplastin time, *Hb* hemoglobin (g/dL), *Hct* hematocrit (%), *MCH* mean corpuscular hemoglobin (pg), *MCV* mean corpuscular volume (fL), *RBC* red blood cells volume (×10^12^ L), *WBC* white blood cells volume (×10^9^ L), *PT* prothrombin time index (%); *p*-value – calculated between A/72 and B/36

The between-animal variability parameters that were observed, expressed the percent of relative standard deviation (RSD%) for baseline, and then separately for the data which were obtained after administration of the drug. For MCV and PT, the RSD% curves obtained after drug administration were lower than or equal to the RSD% obtained for baseline. These values were, for MCV and PT, respectively (baseline:effect), 12.38% vs. 7.26%, and 5.02% vs. 5.02%. For all the other parameters listed in Table 1, the RSD% of baseline ranged from 4.44 to 20.41, and in the case of the curves which covered the effect of RSD%, these ranged from 5.02 to 26.34.

**Fig. 2:**
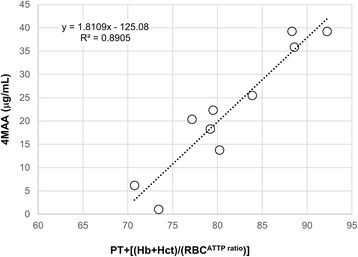
The relationship between the concentration of 4 methylamino antipyrin (4 MAA) and the hemorheological parameter image changes - presented in the form of an arithmetic expression: *PT* + [(*Hb* + *Hct*)/(*RBC* ^*ATT* Pr *atio*^)]. APTT_ratio_ – ratio of activated partial thromboplastin time; Hb – hemoglobin (g/dL); Hct – hematocrit (%); RBC – *red* blood cells volume (× 10^12^ L); PT – prothrombin time index (%)

As presented in the form of an arithmetic expression *PT* + [(*Hb* + *Hct*)/(*RBC* ^*ATT* Pr *atio*^)] (Fig. 2), there was a significant correlation between the concentration of 4MAA and hemorheological parameters. These relationships are statistically significant (*p* < 0.001). The validation parameters that were determined through our work according to the LOO 4MAA↔ *PT* + [(*Hb* + *Hct*)/(*RBC* ^*ATT* Pr *atio*^)] generated the following values: *Q* ^2^_asym_-*Q* ^2^ = 0,063, *Q* ^2^ = 0.8274, *R* ^2^ = 0.8905, *Q* ^2^-*R* ^2^ = 0.0631, SS = 1573.36, PRESS = 271.57. At the same time, our work did not confirm the existence of the same dependency relation to 4AA↔ *PT* + [(*Hb* + *Hct*)/(*RBC* ^*ATT* Pr *atio*^)] (*p* > 0.05).

## Discussion

The aim of the current study was a verification of piglets as a model reflecting changes in hematological parameters after a single I.M. injection of MET. Thrombocytopenia and pancytopenia after MET administration is related to bone marrow suppression. Mechanism covers drug-dependent suppression of the growth of myeloid progenitors, primitive multipotential progenitors and erythroid progenitors [31, 32]. As in humans, the piglet model demonstrates that MET and its metabolites contribute to significant increase of neutrophil count, which represents a large fraction of the total number of WBC. Similar effects were found in rats [33]. In dogs, however, there was no significant MET effect in level of WBC when given at a dose of 25 mg/kg [34]. With regard to piglet model, the effect of the MET on WBC also manifests the fluctuating course of the curve reporting on the effect. Herein, WBC returns to the values obtained with the baseline MET concentration, as well as that of its metabolites, which decline in the blood, 72 h after administration.

What is more, a significant decrease in RBC and Hct after the administration of MET is reported in *Rhamdia sp.* based studies [35]. In addition, in dog-based studies, a decrease in RBC counts were noted after MET administration [4], while in rats, RBC count rose slightly [33]. In the case of proposed piglet model, RBC counts decrease through 72 h after dosing. At the same, no confirmation is evident in this interval for any tendency to return to baseline - as the slope of the curve relating the effect is maintained after 4 h from the administration of the drug, at a constant level up to 72 h. Thus, it confirmed the possibility of utilizing breeding piglets for tracking model purposes in assessing the impact of MET and its metabolites on the physiology of RBC. This process also reflects the decrease in Hb, as the dynamics of changes in relation to the RBC and Hb are similar.

Following administration of MET, in the case of piglets, as well as in humans, Hct levels significantly decrease [36]. Yet, in rat-based studies, MET administration induced a slight fall in Hct [33], while in dogs, no MET-induced effects were seen in Hct levels at a dose of 25 mg/kg [34]. Of note, the observed variability is substantial, however, the observed decrease is significant (*p* < 0.05).

Hb after the administration of MET also has been seen to drop significantly, and the process is characterized by the extremely low volatility of observed data. In both rat and dog-based studies, Hb levels were seen to fall [4, 33]. Regarding piglet model, Hb virtually retains its dynamics between 12 and 72 h after administration.

As shown in the data generated within our study, Hct, RBC and Hb are complementary, and in this case, confirm the predictability of the proposed model. Indeed, between RBC and the sums of Hct and Hb, a strong significant correlation is seen (*R* ^2^ = 0.9500, *p* < 0.05).

In rat-based studies, the value of MCV was seen to drop after MET administration [33]. A similar situation was seen in studies based on utilizing piglets. With regard to MCV + PT, and 4MAA concentrations, up to 48 h after drug administration, a significant linear relationship was evident (*R* ^2^ = 0.8810, *p* < 0.05). In contrast, the same relationship with respect to 4AA was not significant. This implies that MET administration has a direct impact on 4MAA levels (MCV + PT), and, therefore, is essential to the expression of 4MAA.

A single administration of MET (at a dose of 1 g) is known to not significantly affect APTT in humans [37]. However, as shown via the piglet model, a high level of significance regarding MET administration and APTT actually does exist. After 6 h post-dosage, the value of APTT drops significantly. The curve relating the effect of the drug, in this case, reveals the fluctuating course of effect, as between 24 and 72 h post-administration of MET, the value of APTT declines and does not return to the original level. The fluctuating nature of the aforementioned changes may be associated with the impact of MET on the process of plasma recalcification [38], just as administration of MET is recognized as having a relaxation effect on the smooth muscle [39].

In the course of the analysis, we have confirmed the existence of a 4MAA dependence↔ *PT* + [(*Hb* + *Hct*)/(*RBC* ^*ATT* Pr *atio*^)]. This relationship predictive value with respect to 4MAA, confirms the performed validation procedure. This means that hemorheological parameters after administration of MET may reflect the concentration of 4MAA in piglet blood plasma. Of note, the mechanism of the effect of the 4MAA on hemorheological parameters is significantly different from that of 4AA.

## Conclusions

In our study, all the piglets showed tolerance to the dosages given out, and such high doses allowed to analyze precisely the impact of MET on hemorheological parameters. In our study, nine parameters showed significant differences to baseline. Most deleterious effect on blood parameters observed in current study were deep and prolonged decrease of PT and Hb concentration connected with decreased number of RBC. As in humans, the piglet model shown that MET and its metabolites contribute to significant increase of number of neutrophils. The resulting image changes converge to the observations that were noticed in humans. To sum up, our observations suggest that a piglet model is useful for studying MET and its metabolites impact on hemorheological parameters.

## Additional file

Additional file 1: Numerical raw data. (XLSX 20 kb)

## Abbreviations

4AA: 4 aminoantipirin
4MAA: 4 methylaminoantipirin
A: Curve relating the effect after drug administration
APTT_ratio_: Ratio of activated partial thromboplastin time
AUEC: Area under the effect curve
B: Baseline
CT: Closure time
Hb: Hemoglobin
Hct: Hematocrit
I.M: Intramuscular
I.V: Intravenous
LOO: Live-one-out method
MCH: Mean corpuscular hemoglobin
MCHC: Mean corpuscular hemoglobin concentration
MCV: Mean corpuscular volume
MET: Dipyrone
Plt: Platelets
ppm: Pars pro milion
PRESS: Predicted residual sums of squares
PT: Prothrombin time index
*Q*^2^: Squared cross-validated correlation coefficient
*Q*^2^_asym_: Asymptotic squared cross-validated correlation coefficient
R^2^: Squared Pearson correlation coefficient
RBC: Red blood cells volume
RSD%: Relative standard deviation
SS: Total sum of squares
WBC: White blood cells volume
